# 17β-Estradiol and/or estrogen receptor alpha signaling blocks protein phosphatase 1 mediated ISO induced cardiac hypertrophy

**DOI:** 10.1371/journal.pone.0196569

**Published:** 2018-05-03

**Authors:** Hsin-Yuan Fang, Meng-Yu Hung, Yueh-Min Lin, Sudhir Pandey, Chia-Chien Chang, Kuan-Ho Lin, Chia-Yao Shen, Vijaya Padma Viswanadha, Wei-Wen Kuo, Chih-Yang Huang

**Affiliations:** 1 Department of Thoracic Surgery, China Medical University Hospital, Taichung, Taiwan; 2 Graduate Institute of Basic Medical Science, China Medical University, Taichung, Taiwan; 3 Department of Pathology, Changhua Christian Hospital, Changhua, Taiwan; 4 Department of Medical Technology, Jen-Teh Junior College of Medicine, Nursing and Management, Miaoli, Taiwan; 5 Department of Dermatology, Taipei City Hospital, Renai Branch, Taipei, Taiwan; 6 Department of Emergency Medicine, China Medical University Hospital, Taichung, Taiwan; 7 Department of Nursing, Meiho University, Pingtung, Taiwan; 8 Department of Biotechnology, Bharathiar University, Coimbatore, India; 9 Department of Biological Science and Technology, China Medical University, Taichung, Taiwan; 10 Graduate Institute of Chinese Medical Science, China Medical University, Taichung, Taiwan; 11 Department of Health and Nutrition Biotechnology, Asia University, Taichung, Taiwan; San Diego State University, UNITED STATES

## Abstract

Earlier studies have shown that estrogen possess protective function against the development of pathological cardiac hypertrophy. However, the molecular mechanisms of estrogens (E2) protective effect are poorly understood. Additionally, abnormal activation of β-adrenergic signaling have been implicated in the development of pathological cardiac remodeling. However, the role of serine/threonine protein phosphatase 1 (PP1) in pathological cardiac remodeling under the influence of β-adrenergic signaling have been sparsely investigated. In this study, we assessed the downstream effects of abnormal activation of PP1 upon isoproterenol (ISO) induced pathological cardiac changes. We found that pre-treatment of 17β-estradiol (E2), tet-on estrogen receptor-α, or both significantly inhibited ISO-induced increase in cell size, hypertrophy marker gene expression and cytosolic calcium accumulation in H9c2 cells. Additionally, treatment with estrogen receptor inhibitor (ICI) reversed those effects, implicating role of E2 in inhibiting pathological cardiac remodeling. However, specific inhibition of ERα using melatonin, reduced ISO-induced PP1c expression and enhanced the level of ser-16 phosphorylated phospholamban (PLB), responsible for regulation of sarcoplasmic reticulum Ca^**2+**^-ATPase (SERCA) activity. Furthermore, hypertrophic effect caused by overexpression of PP1cα was reduced by treatment with specific inhibitor of ERα. Collectively, we found that estrogen and estrogen receptor-α have protective effect against pathological cardiac changes by suppressing PP1 expression and its downstream signaling pathway, which further needs to be elucidated.

## Introduction

Estrogen is one of the steroid hormones produced mainly by ovaries, less in testicles and adrenal cortex. Estrogen exists in three different isoforms: estrone (E1), 17β-Estradiol (E2) and Estriol (E3), respectively. E2 is considered to be most abundantly produced among all three. The functions of E2 include modulation of cell development and differentiation, development of reproductive system, maintenance of bone integrity, protection of vascular system and regulation of central nervous system [1]. Clinical research has shown that women exhibited lower risks of developing cardiac hypertrophy compared to the men of same age. After entering menopause, the rate of cardiac hypertrophy increased significantly among postmenopausal women. The studies showed that hormone replacement therapy was effective in relieving the symptoms cardiac hypertrophy [2,3]. The same phenomenon was observed in animal experiment, hormone replacement therapy significantly inhibited the development of cardiac hypertrophy in ovariectomized rats [4]. The evidence suggested that female hormone exhibited a protective role in cardiovascular system by inhibiting abnormal cardiac structural changes. However, the underlying cardioprotective mechanisms are yet to elucidated. Furthermore, the intracellular calcium levels largely regulate sensitive cellular proteins associated with cardiac anomalies. The animal experiments have shown that estrogen protected FKBP12.6 (a protein associated with calcium release and regulation in sarcoplasmic reticulum) null mice from cardiac hypertrophy [5]. In clinical studies, it was evident that heart disease was highly associated with imbalance of calcium influx, especially in end stage heart failure patients [6,7]. However, cardiac hypertrophy is thought to be a compensatory mechanism in response to acute or chronic damages to heart. On the other hand, prolonged stimulus dependent hypertrophy causes cardiac overload which raises potential risks to develop a heart failure [8]. Abnormal activation of β-adrenergic receptor leads to phosphorylation of L-type calcium channel and RYR2 channels located in sarcoplasmic reticulum (SR), respectively and release of calcium from intracellular calcium stores [9,10]. Moreover, disassociation of a regulatory protein, FKBP12.6, from the phosphorylated RYR2 protein complex compromised the integrity of its tetramer structure resulting in leakage of calcium from SR. However, de-phosphorylation of PLB inhibited sarcoplasmic reticulum Ca^**2+**^-ATPase (SERCA) activity which lowered the rate of calcium recycling in cytosol [11,12]. The over accumulation of calcium not only destroyed cell contraction ability but also activated calcineurin (protein phosphatase 2B; PP2B) [13] which de-phosphorylated and activated transcription factor, NFAT3. Subsequently, the activated NFAT3 lead to transcription of cardiac hypertrophy associated genes, for example atrial natriuretic peptide (ANP), brain natriuretic peptide (BNP) and skeletal muscle actin (SkMu). Finally, the activation of calcium mediated cardiac hypertrophic signaling pathway lead to development of heart diseases [8]. In heart, the two main Ser/Thr protein phosphatases (PP1 and PP2A) are modulated by intracellular calcium fluctuations [14]. Clinical studies have revealed that both PP1 gene and protein expressions were elevated and its downstream proteins, PLB and troponin (Tn) were de-phosphorylated in heart tissues collected from heart failure patients [15,16]. As mentioned previously, β-adrenergic receptor which regulated PP1 protein activation was highly activated in heart failure patients. Earlier studies by Neumann group suggested the link between β-adrenergic receptor, PP1 and heart failure, using Isoproterenol (ISO) induced β-adrenergic receptor stimulation in animal model. They showed that chronic ISO stimulation lead to enhanced expression of both PP1 and PP2A followed by de-phosphorylation of PLB gradually resulting in development of cardiac hypertrophy [17]. Further studies showed that over expression of either PP1 [18] or PP2A [19] resulted in cardiac hypertrophy and malfunction of heart which subsequently lead to progression of heart diseases. These studies implicated the involvement of PP1 as one of the causal factors for the development of heart failure. Several therapeutic attempts including inhibition of PP1, by inhibitor-1 (I-1) (a protein inhibitor of PP1), or inhibitor-2 (I-2) [20] were carried out in order to prevent deterioration of heart under cardiac stress conditions [21,22]. As such, further studies were warranted to understand the underlying mechanisms for the involvement of PP1 in the development of cardiac anomalies and we attempted through this study to elucidate the role of PP1 in estrogen mediated cardiac protection upon ISO induced pathological cardiomyocyte enlargement.

## Materials and methods

### Cells, antibodies, reagents, and enzymes

H9c2 cells were obtained from the American Tissue Culture Collection and cultured in Dulbecco’s modified Eagle’s medium (DMEM) supplemented with 100 μg/ml penicillin, 100 μg/ml streptomycin, 2 mM glutamine, 1 mM HEPES buffer, and 10% HyClone fetal bovine serum in humidified air (5% CO2) at 37°C. Tet-on/ERα H9c2 cardiomyoblast cells were generated as described previously [23]. Rhodamine–phalloidin was purchased from Molecular Probes (Eugene, Oregon, USA). The ER antagonist ICI 182780 (ICI) was purchased from Tocris (Ellisville, Missouri, USA). All the chemicals used were purchased from Sigma Aldrich (St. Louis, Missouri, USA) unless stated otherwise. α-tubulin antibodies was purchased from Lab Vision Corporation (Fremont, California, USA), protein phosphatase 1 (PP1cα), phosphorylated PLB (p-PLB) and PLB were obtained from Upstate (Lake Placid, NY, USA). Secondary antibodies were obtained from Santa Cruz Biotechnology (Santa Cruz, California, USA).

### Construct Tet-on gene expression system

Tet-on Gene Expression System that uses two different expression plasmids to cooperatively control gene expression was employed. Plasmid pTet-on, constitutively expressed rtTA protein which binds to the promoter of pTRE plasmid and activates it along with Dox or Tet. Tet-on/ERα H9c2 cardiomyoblast cells were generated as previously described [23].

### Actin staining

Tet-on/ERα H9c2 cells were fixed with 4% paraformaldehyde, permeabilized with 0.5% Triton X-100, and blocked with 1% BSA in phosphate-buffered saline (PBS) for 10 min. Rhodamine-labeled phalloidin (red) staining was performed to visualize actin filaments as described previously [24]. Cells were examined microscopically to ascertain morphological changes and the cell area was measured using Carl Zeiss AxioVision LE software. Surface area was quantified by imaging to the complete boundary of individual cells in various conditions and thirty cells in each condition were counted and regarded as an independent experiment. More than 10 fields for each condition were analyzed and quantified from three independent experiments.

### Intracellular calcium staining

Ca2+ staining was performed according to the manufacturer’s protocol which has been reported earlier [25]. Fluorescence was monitored at 528 nm (excitation: 490–500 nm) and images were acquired using a fluorescence microscope.

### Transient transfection

Cell transfection was performed using Lipofectamine 2000 transfection kit (Invitrogen, USA) according to manufacturer’s instructions and the cells were harvested at the end of indicated time points.

### Total RNA extraction and reverse transcription reaction (RT)

Total RNA was extracted using the Ultraspec RNA isolation kit (Biotecx Laboratories, Houston, Texas, USA) according to protocol recommended by the manufacturer. For RT-PCR, 4 μg of total RNA was reverse transcribed (RT) using SuperScript III reverse transcriptase (Invitrogen) kit. RT was carried out at 42°C for 30 min followed by incubation at 95°C for 5 min. cDNA amplification was carried out under the temperature profile: 94°C for 30 s; 55°C for 30 s; and 72°C for 1 min. After 35 cycles, final amplification was carried out for 7 min [26]. List of primers used have been mentioned in Table 1.

**Table 1:** Primers used in the study.

Gene	Primer sequence	Amplicon size (bp)	Temperature (°C)	Cycle
Rat skeletal muscle actin (SkMu Actin)	5' CACGGCATTATCACCAACTG 3' TCTCACGTTCAGCTGTGGTC	406	58	28
Rat ANF	5' TGCCGGTAGAAGATGAGGTC 3' ATTCACCACCTCTCAGTGGC	403	59	32
Rat BNP	5' TTTTCCTTAATCTGTCGCCG 3' CACTGTGGCAAGTTTGTGCT	498	57	32
Rat PP1c	5' TTAGACGTATTATGCGGCCC 3' CTTAACGCTGTGTTCCCCAT	549	58	28
GAPDH	5' GGGTGTGAACCACGAGAAAT 3' CCACAGTCTTC TGAGTGGCA	167	58	25

ANF, Atrial Natriuretic Factor; BNP, Brain Natriuretic Peptide; PP1c, Protein Phosphatase 1c; GAPDH, Glyceraldehyde 3-phosphate dehydrogenase

### Western blotting

Proteins were separated using 10–12% SDS-PAGE and then transferred to PVDF membranes for 1.5 h at 90 V. PVDF membrane was then incubated in blocking buffer.

(5% milk in 1xTBS) for 1 h at room temperature. The membranes were incubated with the primary antibodies at the recommended concentrations at 4°C overnight and then incubated with the secondary antibodies for 1 h at room temperature [27,28]. The results were visualized with chemiluminescent HRP substrate (Millipore, MA, USA).

### Statistical analysis

In this study, statistical analysis was performed using student’s t-test, and significance was set at p <0.05. The results shown represent the mean ± SD from three independent experiments.

## Results

### ISO treatment increased cell size in Tet-on/ERα H9c2 cells in a time course assessment which was reversed by ERα inhibitor

**Fig 1:**
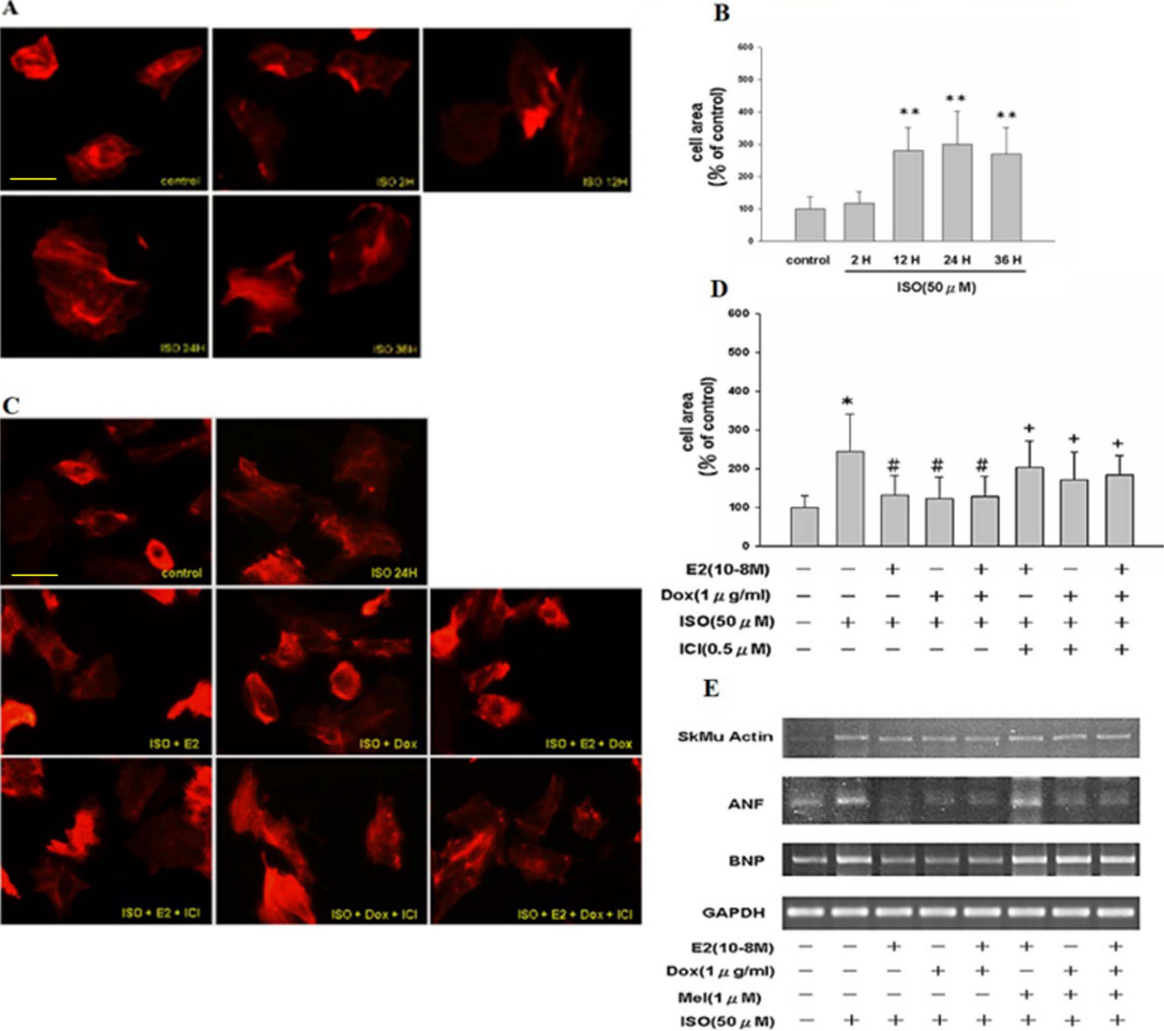
ISO treatment leads to hypertrophic cellular morphology in H9c2 cardiomyocytes in a time-dependent manner. **A**, Fluorescence images of Tet-on/ERα H9c2 cells treated with ISO for 2 h, 12 h, 24 h and 36 h, respectively. As evident from actin staining, the cell surface area was enhanced after ISO stimulation in a time dependent manner. **B**, Quantification of cell surface area from above experiment. The bar graph represents data from three independent experiments and shows mean ± S.D. Student’s t–test was performed to arrive at significance. “**”, p < 0.01 for control versus ISO. **C**, Tet-on/ERα H9c2 cells were incubated with ISO, ISO plus E2 or Dox or ICI or combined treatments for 24 hours and actin staining was performed. E2 or Dox or combined treatment reduced the cell surface area, which was negated by ICI treatment. **D**, Tet-on/ERα H9c2 cell surface area from above experiments was quantified. The bar graph (mean ± S.D. of response) represents data from three independent experiments. 30 cells were counted per condition in each experiment. “*”, p < 0.05 for control versus ISO; “#”, or “+”, p *<* 0.05 for ISO versus combined treatments. **E**, Cells were incubated with ISO, ISO plus E2 or Dox or melatonin or combined treatments for 24 h. E2, Dox or both inhibit ISO-induced skeletal muscle actin, ANF, and BNP gene expression as measured by RT-PCR analysis, which was reversed by melatonin treatment (specific ERα inhibitor).

Earlier studies have shown that ISO treatment leads to hypertrophy in H9c2 cells. In order to further elucidate the underlying molecular mechanisms, we treated Tet-on/ERα H9c2 cells with isoproterenol (ISO, 50μM) for 2 h, 12 h, 24 h and 36 h, respectively. Using rhodamine-conjugated phalloidin to detect the actin filaments, we found that H9c2 cells exhibited hypertrophic cellular morphology (Fig 1A and 1B) upon ISO treatment in a time dependent manner. To further understand the role of estrogen signaling in ISO induced cardiac hypertrophy, Tet-on/ERα H9c2 cells were pre-treated with E2 and/or Dox in the presence or absence of ISO for 24 h. We found that pre-treatment with E2, Dox, or E2 plus Dox significantly inhibited ISO-induced hypertrophic changes in cardiomyoblast cells. To further ratify the role of E2 and/or ERα in suppressing ISO-induced hypertrophic cellular growth, cells were treated with ER inhibitor (ICI), which effectively antagonized the effects of E2 and/or ERα in ISO-treated H9c2 cells (Fig 1C and 1D). Next, to consolidate above observations, Tet-on/ERα H9c2 cells were pre-treated with E2 or Dox or melatonin (a specific inhibitor of ERα) in the presence or absence of ISO for 24 h. Using RT-PCR analysis, we found that mRNA expression of cardiac hypertrophy associated markers ANF, BNP and SkMu actin were significantly inhibited by E2, Dox and/ or combination treatments. However, treatment with specific ERα inhibitor melatonin reversed the anti-hypertrophic effects mediated by E2 treatment (Fig 1E). Above findings indicate that ISO-induced hypertrophic effect in H9c2 cells could be significantly attenuated by E2 and/or ERα treatment.

### ISO-induced intracytoplasmic calcium accumulation in H9c2 cardiomyoblast cells was reduced by E2 and/or ERα

**Fig 2:**
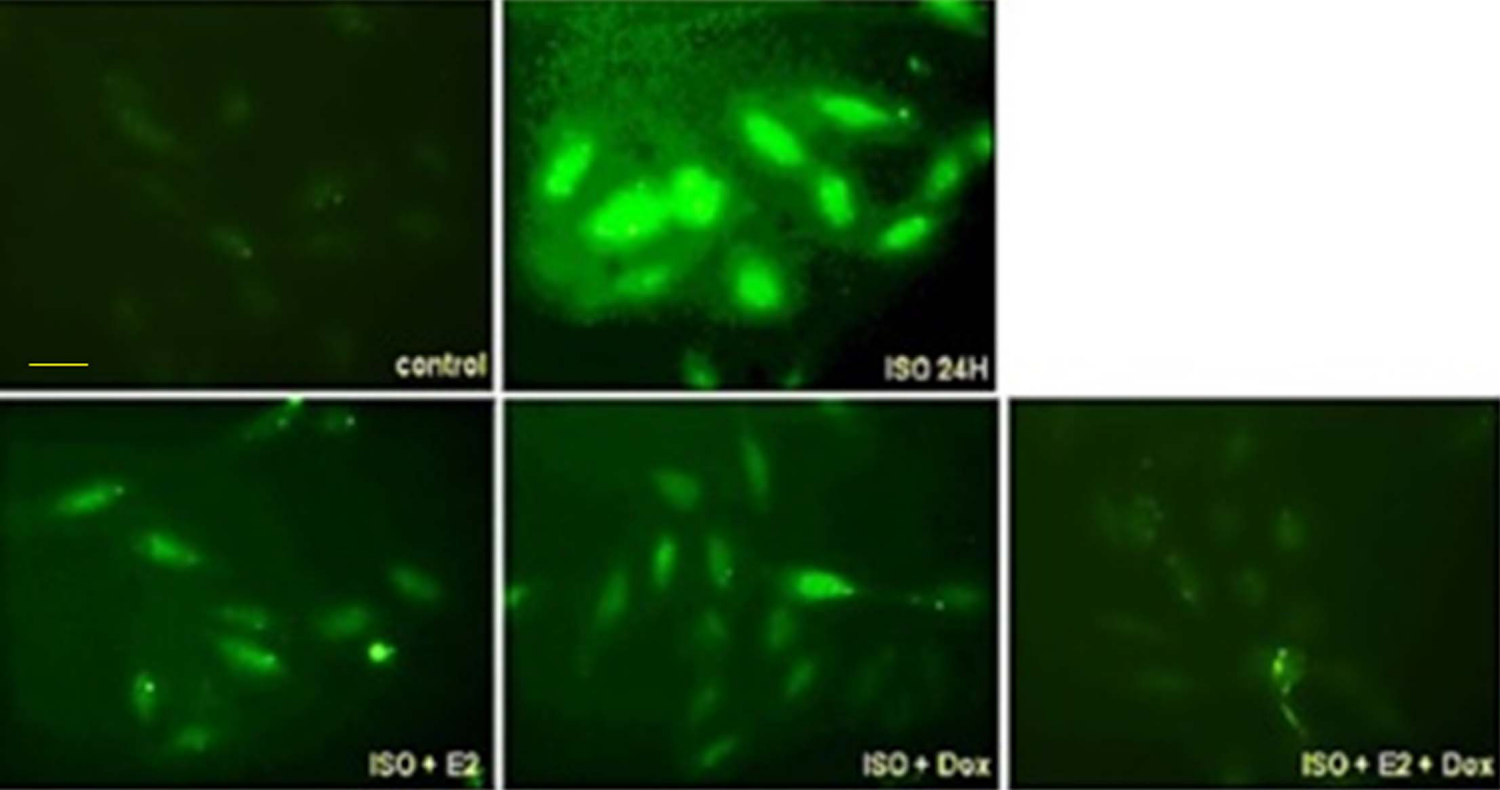
ISO-induced calcium accumulation in H9c2 cardiomyoblast cells was reduced by E2 and/or ERα. Tet-on/ERα H9c2 cardiomyoblast cells were incubated with E2 and/or Dox in presence or absence of ISO for 24 h, and followed by calcium staining. The intracytoplasmic calcium accumulation upon ISO induction was reduced by E2 and/ or Dox treatment as visualized by fluorescent microscope.

Earlier studies have demonstrated cytoplasmic accumulation of calcium upon ISO treatment in H9c2 cells which activate the calcium sensitive cellular proteins and downstream cardiac hypertrophic pathways. To further elucidate the underlying molecular mechanisms, we pre-treated Tet-on/ERα H9c2 cells with estrogen and/or Dox in presence or absence of ISO for 24 h. At the end of treatment, calcium staining was performed and we found that ISO treatment for 24 h induced intra-cytoplasmic calcium accumulation. However, pre-treatment by estrogen and/or Dox effectively reduced ISO-induced cytoplasmic calcium accumulation (Fig 2).

### PP1cα expression was enhanced by ISO treatment, however the effect was reversed by E2 or Dox and/ or melatonin treatment

**Fig 3:**
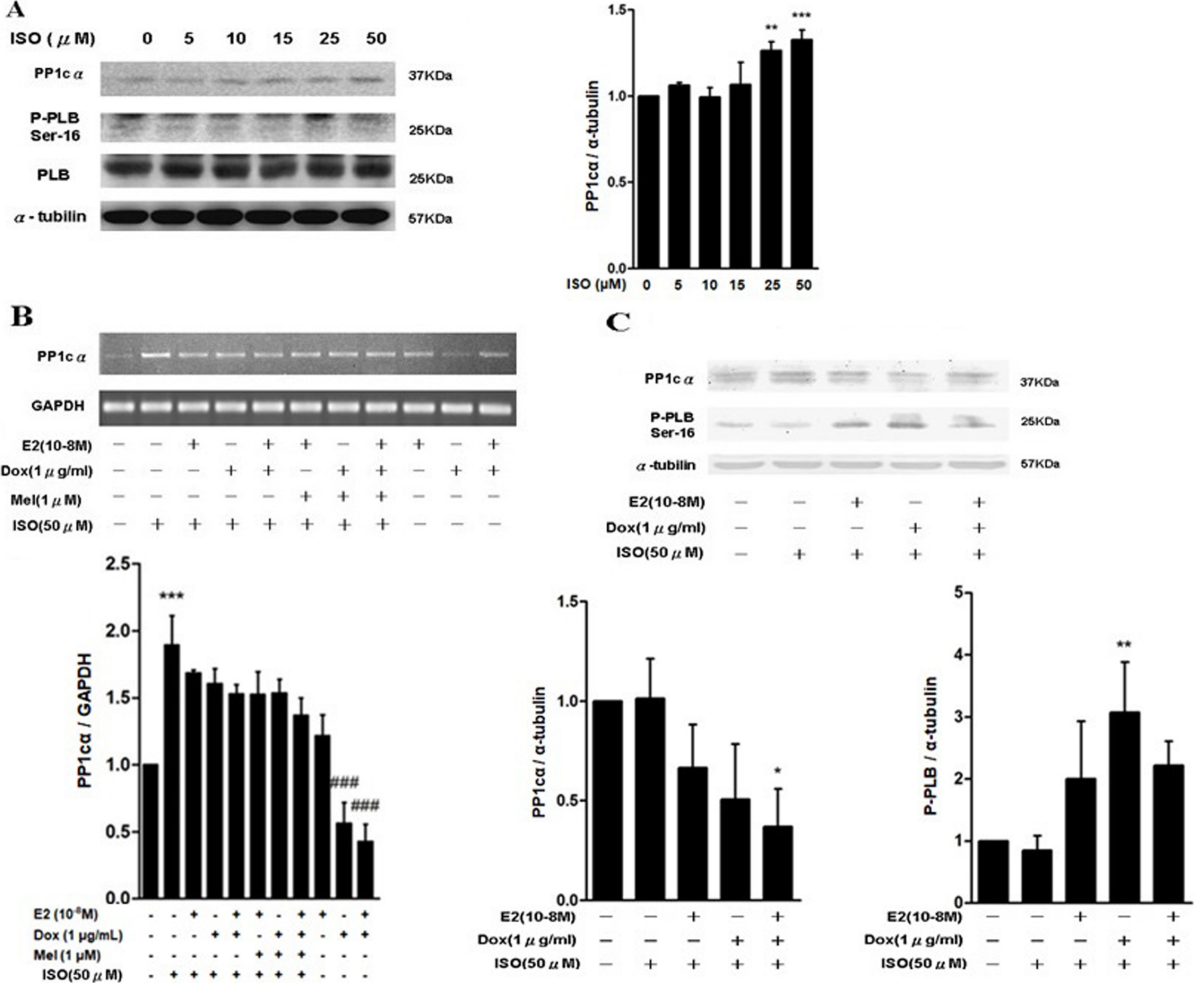
PP1cα expression was enhanced by ISO treatment, which was reversed by E2 or Dox and/ or melatonin treatment. **A,** Tet-on/ERα H9c2 cells were incubated with different concentrations of ISO for 24 h. Western blot analysis showed that protein expression level of PP1cα was enhanced while Ser16 phospho-PLB protein was decreased in a dose dependent manner. However, protein expression of unphosphorylated PLB did not change significantly in presence of ISO alone. “**”, p < 0.05 for ISO versus control; “***”, p < 0.01 for ISO versus control. **B & C,** Tet-on/ERα H9c2 cells were pre-treated with either E2 or Dox or melatonin or combination treatments with or without ISO for 24 h. PP1cα expression was inhibited by E2 or Dox or melatonin treatment at both transcriptional level as determined by RT-PCR (“***”, p < 0.05; “###”, p< 0.05 versus untreated) and translational level (“*”, p < 0.05; “**”, p < 0.01) by western blot analysis, respectively.

Previous studies have indicated that treatment with β-adrenergic receptor agonists enhanced PP1 activity in cardiac ventricular myocytes [29,30]. Therefore, we investigated PP1cα expression in ISO treated H9c2 cells. We found that protein expression level of PP1cα was enhanced while Ser16 phospho-PLB protein was decreased in a dose dependent manner. However, protein expression of unphosphorylated PLB was unchanged (Fig 3A). To understand further the effect of E2 signaling over ISO induced PP1c expression, Tet-on/ERα H9c2 cells were pre-treated with E2, Dox, melatonin or combination treatments with or without ISO for 24 h. We found that ISO induced PP1c expression was inhibited by E2 or Dox or melatonin treatment at both transcriptional and translational level. Above results were further confirmed by western blot analysis (Fig 3B and 3C). Based on the above findings, we concluded that E2/ERα signaling pathway is involved in regulating expression of PP1cα during ISO induced cardiomyocyte hypertrophy.

### PP1cα overexpression induced cardiomyoblast hypertrophy which was alleviated by E2 and/ or Dox treatment, while the effect was reversed by treatment with specific inhibitor of ERα

**Fig 4:**
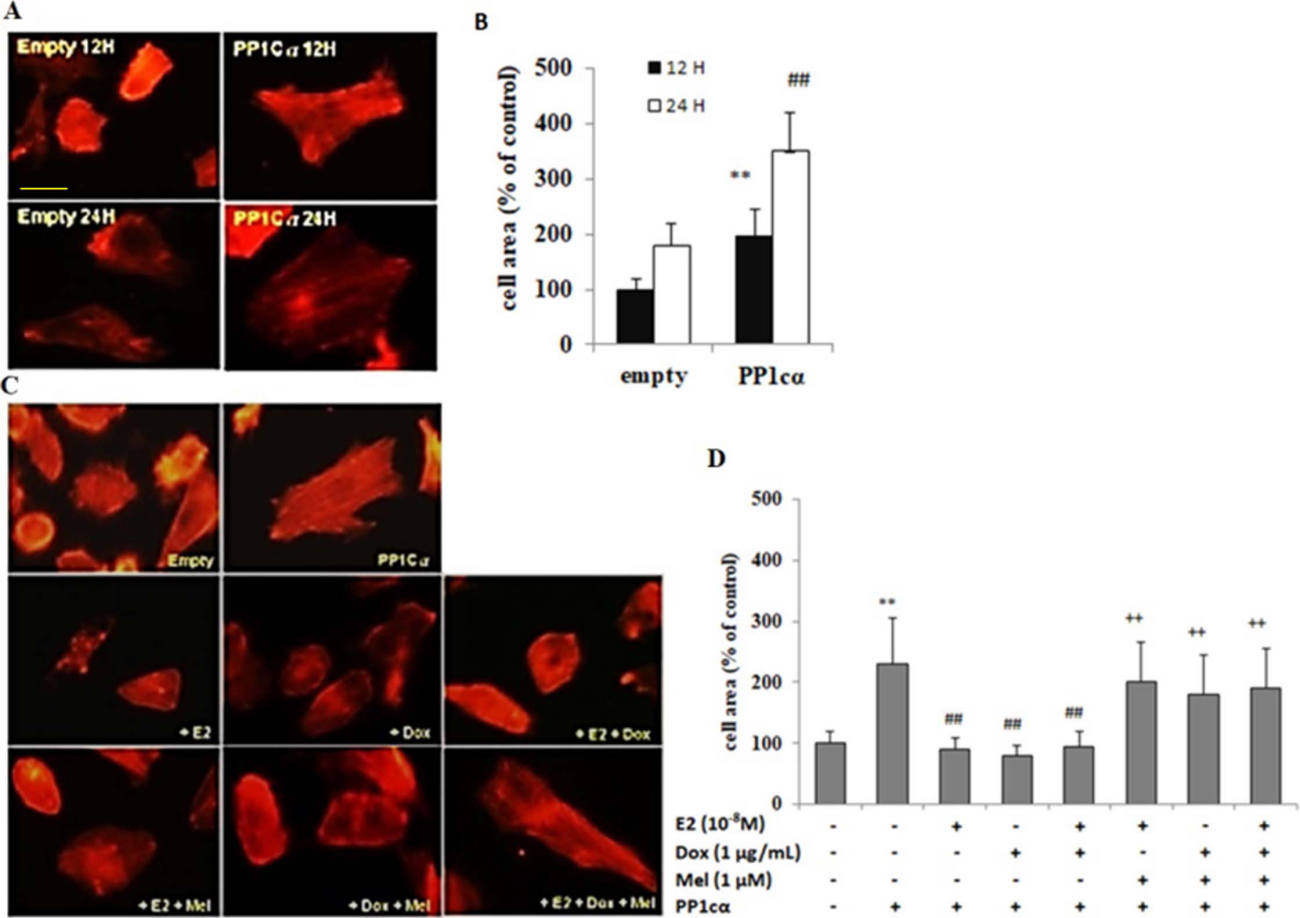
PP1cα overexpression mediated cardiomyoblast hypertrophy was attenuated by E2 and/ or Dox treatment, which was reversed by treatment with specific ERα inhibitor. **A & B**, Tet-on/ERα H9c2 cells were transfected with overexpressing vectors carrying PP1cα. At 12 h, actin staining was performed which showed that cell surface area was increased by 2 folds in PP1cα transfected cells as compared to cells transfected with empty vectors alone. At 24 h, further increase in cell surface area was observed by approximately 1.5 folds as compared to 12 h and cell surface area quantification was performed. Student’s t-test, “**”, p < 0.01 for control versus PP1cα 12 h; “##”, p < 0.01 for control versus PP1cα 24 h. **C & D**, Tet-on/ERα H9c2 cells were pre-treated with either E2 or Dox, or melatonin or combination treatments, followed by transfection with PP1cα overexpressing vectors for 24 h. As evident by actin staining, E2 or Dox or combination treatments reduced hyper growth effect induced by PP1cα overexpression. However, treatment with melatonin reversed the anti-hypertrophic effect of E2, Dox and/ or combination treatments in PP1cα transfected cells. The bar graph (mean ± S.D.) represents data from three independent experiments. 30 cells were counted per condition in each experiment, and the data was analysed by Student’s t-test. “**”, p < 0.05 for untreated versus PP1cα; “##”, p < 0.05 versus PP1cα or “++”, p < 0.01 versus untreated.

To further understand the role of PP1cα in ISO induced cardiac hypertrophy, Tet-on/ERα H9c2 cells were transfected with overexpressing vectors carrying PP1cα. At 12 h, actin staining was performed which showed that cell surface area was increased by 2 folds in PP1cα transfected cells as compared to cells transfected with empty vectors alone. At 24 h, further increase in cell surface area was observed by approximately 1.5 folds as compared to 12 h (Fig 4A and 4B). In contrast, either E2, Dox or combination treatments reduced hyper growth effect induced by PP1cα overexpression. However, treatment with melatonin reversed the anti-hypertrophic effect of E2, Dox and/ or combination treatments in PP1cα transfected cells (Fig 4C and 4D). Collectively, our study demonstrated that ISO induced cardiac hypertrophic growth involves decrease in Ser-16 phosphorylated PLB by increased activation of PP1c. However, estrogen signaling displayed preventive and protective effect against hypertrophic changes by suppressing PP1c expression and enhancing Ser-16 phosphorylated PLB expression and inhibiting intracytoplasmic accumulation of calcium.

## Discussion

**Fig 5:**
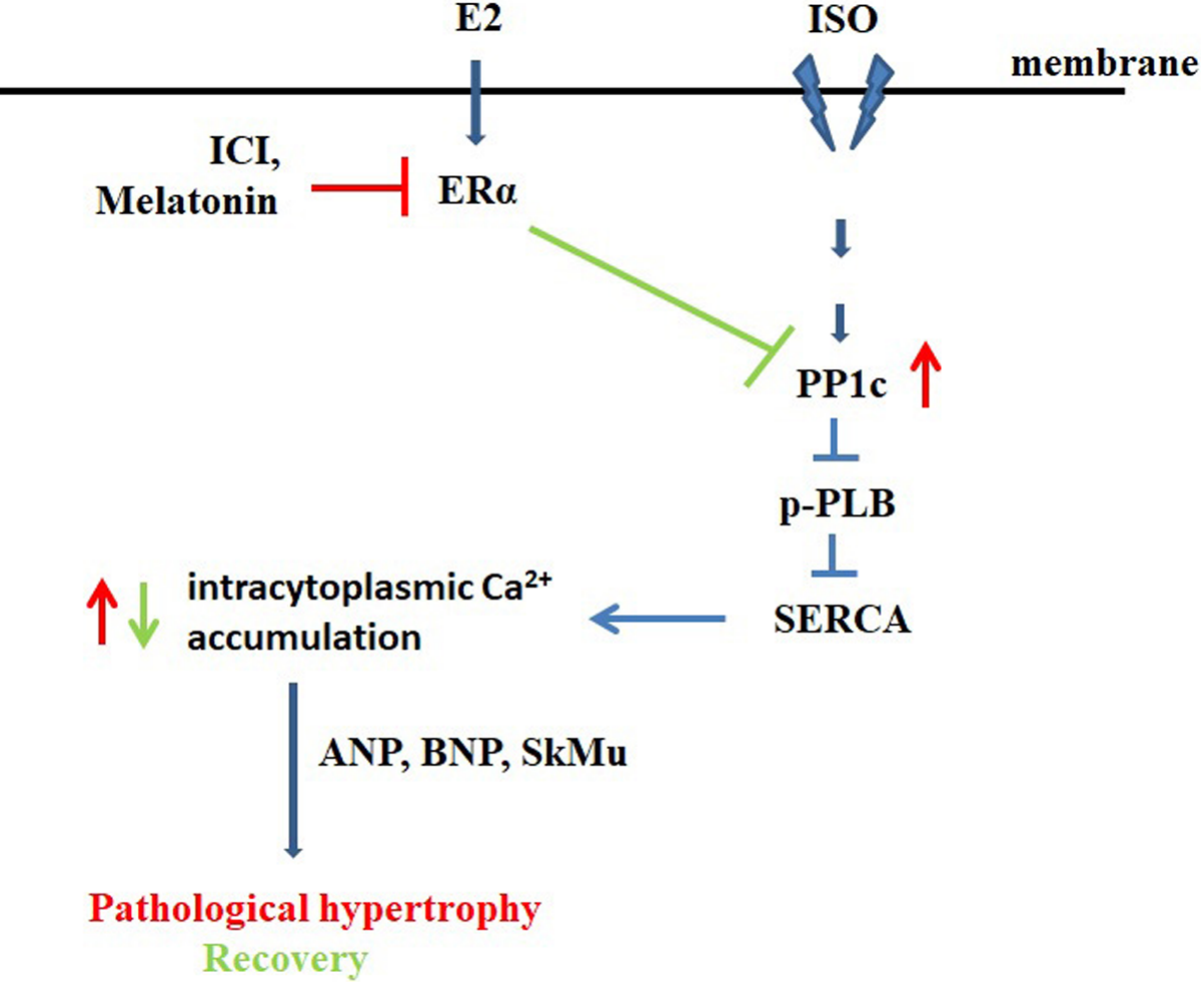
Graphic summary. In ISO-induced cardiomyoblast hypertrophy model, we demonstrated that ISO treatment enhanced intracytoplasmic calcium accumulation which was correlated with increased PP1cα expression and E2 treatment suppressed the hypertrophic effect caused by ISO treatment. However, inhibiting E2 signaling by ICI or melatonin treatment resulted in significant loss of protective anti-hypertrophic effect. Furthermore, we showed that ISO induced cardiomyocyte hypertrophy was mediated by PP1cα over-expression and E2 treatment was sufficient to block PP1cα expression and exert protective effect against hypertrophy induced by ISO treatment.

Much evidence through earlier studies indicate that over stimulation of β-adrenergic receptor lead to up-regulation of protein phosphatase 1 and 2A, which contribute to cardiac hypertrophy [31]. Other evidences in this line showed that estrogen signaling exerts its protective effect against cardiotoxicity through suppressing pathways such as such as calcineurin/NFAT3 and the JNK1/2-NFkB pathways [23,32]. In this study, we established ISO-induced Tet-on/ERα H9c2 cell model to induce cardiomyoblast hypertrophy in vitro and investigated the role of E2 and/ or ERα against ISO induced pathological cardiac hypertrophy. We found that ISO treated cells markedly became larger in a time-dependent manner in Tet-on/ERα H9c2 cells. Earlier studies have shown that mechanical stress induces expression of cardiac hypertrophic markers such as ANF, BNP and skeletal alpha-actin in neonatal ventricular cardiomyocytes and atrial preparations [33], as such we measured the expression level of these markers in H9c2 cells. We demonstrated that both E2 and/ or ERα pre-treatment inhibited ISO-induced cardiomyoblast hypertrophy and cytosolic calcium accumulation in cells. While, the anti-hypertrophic effect by E2 was lost by ER inhibitor ICI or melatonin treatment. The effect of E2 and/ or ERα on PP1c expression and its downstream effect over cardiomyocyte hypertrophy were also investigated. We demonstrated that cardiomyocyte hypertrophy induced by ISO mediated PP1c overexpression was significantly attenuated by E2 treatment; however, the effect was blocked by specific ER inhibitor melatonin treatment. Thus, we concluded that the inhibitory effect of E2 and/ or ERα on ISO-induced hypertrophy was mediated through interfering with PP1c protein expression and its downstream signaling. Previous reports have shown that E2 inhibited AngII-induced cardiac hypertrophy through various mechanisms [34]. Most importantly, E2 could up regulate modulatory calcineurin-interacting proteins (MCIP1) expression that deactivate calcineurin [35] and nuclear translocation of transcription factor, NFAT3, is inhibited, resulting in blockage of the transcription and translational of cardiac hypertrophic genes. In ISO-induced cardiomyoblast hypertrophy model, we demonstrated that ISO treatment enhanced intracytoplasmic calcium accumulation which was correlated with increased PP1c expression and E2 treatment suppressed the hypertrophic effect caused by ISO treatment. However, inhibiting E2 signaling by ICI or melatonin treatment resulted in loss of anti-hypertrophic effect. Further, we showed that ISO induced cardiomyocyte hypertrophy was mediated by PP1c over-expression and E2 treatment was sufficient to exert protective effect against hypertrophy induced by ISO treatment (Fig 5). However, further investigations are warranted to delineate downstream signaling pathways such as calcium/NFAT signaling and potential regulators which can modulate the development of cardiac remodeling process during ISO induced cardiac pathological changes.
